# The experience of nurses in care for culturally diverse families: A qualitative meta-synthesis**^1^**

**DOI:** 10.1590/1518-8345.1052.2718

**Published:** 2016-07-04

**Authors:** Saidy Eliana Arias Murcia, Lucero Lopez

**Affiliations:** 2 RN, MSc, Doctoral student, Universidade Federal de Minas Gerais, Belo Horizonte, MG, Brazil.; 3 PhD, Associate Full, Facultad de Enfermería, Universidad Nacional de Colombia, sede Bogotá, Colombia.

**Keywords:** Review, Family Nursing, Cultural Diversity, Transcultural Nursing

## Abstract

**Objective:**

to understand the experience of nurses in care delivery to culturally diverse families.

**Method:**

qualitative meta-synthesis. Exhaustive search in seven databases, three repositories and a manual search in references without time limit, in English, Spanish and Portuguese, resulting in 1609 potentially relevant studies. These were assessed based on the title, summary and full text, determining the final inclusion of 14 studies. Two independent reviewers used the Critical Appraisal Skills Programme (CASP) to assess the quality. The interpretative synthesis implied permanent contrast and consensus among the authors, revealing four categories and one meta-theme.

**Results:**

"taking care of a culturally diverse family, the experience of crossing a tightrope".

**Conclusion:**

the experience of nurses in care delivery to culturally diverse families is demanding and challenging because it imprints a constant tension among barriers, cultural manifestations and the ethical responsibility of care, incipiently revealing elements of cultural competency. The omission of information in the participants' reports in the studies represents a limitation. The findings offer a baseline for professionals and organizations to focus their intervention efforts on the continuing barriers in care delivery to culturally diverse families and strengthens the need for cultural competency training for nurses.

## Introduction

The cultural diversity of multicultural societies results from the coexistence of different ethnic groups in the same country or region^1^; from the differences between and within the same groups or regions^2^; the growth of migration flows^3^; the social class, education, gender, language, age, religion and family structure^4^ ^-^ ^5^.

In Latin America, white people, indigenous people of different ethnic origins, afro descendants, mulattos, people of mixed origins, European and Asian migrants live together; five official languages are spoken (Spanish, Portuguese, French, English and Guaraní), as well as 420 indigenous languages belonging to 99 linguistic families^6^. Therefore, one cannot refer to "Latinos" as a homogeneous cultural group, but the cultural diversity needs to be considered.

This cultural multiplicity confronts the health area with particular demands, not only to cope with the inequalities, promoting services in accordance with the cultural singularity^7^, but also to overcome the individual focus to deliver culturally competent care. Attending to the families' preferences has shown to be a relevant strategy, applied to enhance the effect of culturally competent interventions^8^ ^-^ ^11^, as it reflects an understanding of the beliefs and traditions^8^ and takes into account the ethnic, cultural and socioeconomic plurality characteristic of the multicultural groups^10^ ^,^ ^12^.

Thus, health professionals need to feel committed to the delivery of culturally competent care, turning into a critical and essential factor in health care provision to families of all racial, ethnic and cultural origins^13^ ^-^ ^14^. Nevertheless, putting such actions in practice remains demanding, due to the existence of barriers^12^ associated with personal and contextual factors that can facilitate or impede the care^15^.

A metasynthesis on the experiences of nurses in care delivery to patients from other cultures^16^ reported on the nurses' concern with communication barriers, access opportunities and care quality; benefits of learning on other cultures and satisfaction in care delivery to patients of different ethnic origins. Based on these findings, the author suggested the conception of new meta-syntheses with more specific approaches of care delivery scenarios, cultural groups and care practices to facilitate direct comparisons^16^.

The growing production of qualitative studies on the experience of nurses involving patients of different cultural origins has highlighted findings on the family-centered culturally competent care experience, which have not been synthesizes thus far to support evidence-based practice.

To fill this void, this review was proposed based on a meta-synthesis as an interpretive product and analytic process, aimed at interpreting and synthesizing the findings of qualitative studies to understand the experiences of nurses in care delivery to culturally diverse families.

For the sake of this review, culturally diverse families were considered as family members of an adult person over 18 years of age from ethnic origins, countries or religions different from the nurse's.

In addition, in this study, family care was considered in a broad sense, considering the family as a care unit, system or context for the patient^17^, with a view to including all approaches of the family in the care environment. The nurses' experience was conceived as anything (thoughts, feelings, reflections and actions) witnessed, felt or recalled, described by the nurses during care for culturally diverse families^18^.

In the particular case of Latin American countries, the knowledge development resulting from this synthesis contributes to the production of answers to the health demands of culturally diverse families, professional training in cultural competency, and the adaptation of family intervention programs and policies that permit guaranteeing safety, quality and care compliance.

## Method

Qualitative meta-synthesis with interpretive focus, according to the parameters suggested by Sandelowski and Barroso^19^. In this interpretive integration, the results of qualitative studies on the experiences of nurses in care delivery to culturally diverse families were aggregated, integrated and summarized to creative interpretive representations in a new result that is faithful to the interpretation of the particular studies^19^.

This process implies the design of a review protocol proposed during a research training experiences the first author undertook in April 2014 in Sao Paulo, Brazil.

The definition of the question was guided by the PICo strategy, which JBI suggests for qualitative systematic reviews. This strategy permits the specification of key aspects related to the population (P), phenomenon of interest (I) and context (Co)^20^, defined as follows: What has been the experience of nurses in care delivery to culturally diverse families?

The search strategy was proposed under the advice of specialized librarians from the University of Sao Paulo and implemented between April 14^th^ and 18^th^ 2014. The consulted databases included CINAHL, Medline, Ovid Nursing, Science Direct, Sociological Abstracts, Cuiden, BVS-Lilacs and the Repositories of the Universidad Nacional de Colombia, Universidad de Alicante (Spain) and the University of São Paulo (Brazil).

The controlled search terms (MeSH and Decs) and key words for the search in English were: "Attitude of Health Personnel", "Nurse-Patient Relations", "Nurse experience", "Family Nurse Practitioners", "Professional-Family Relations", "Family Centered Nursing", "Culturally diverse families" and "Multicultural family members" and their equivalent in Spanish and Portuguese. These terms were specified according to the thesaurus tools by each database and integrated using the Boolean operators "AND" and "OR", according to the parts of the PICo question.

In addition, a manual search was undertaken in the references of the studies included, with a view to reducing the publication and selection bias in the exclusion of the literature within the range of the review^21^.

The following inclusion criteria were considered to select the studies: a) English, Spanish and Portuguese, without time limit, published until March 30^th^ 2014; b) reports of findings on the experiences of nurses with culturally diverse families of adults over 18 years of age in any care sphere and c) ethnographic, phenomenological designs, grounded theory, discourse analysis, oral life history, participatory action-research and mixed studies with possibilities to extract qualitative data.

As a result of this search phase, 1,621 reports were obtained, 12 of which were repeated. Hence, in total, 1,609 reports were submitted to the selection process per title, abstract and full text based on the inclusion criteria. In addition, the manual review of the references revealed five reports. That resulted in the inclusion of a final sample of 14 studies for this meta-synthesis^22^ ^-^ ^35^.

To assess the quality of the studies, the Critical Appraisal Skills Programme (CASP) for qualitative studies was used, involving two independent teams of reviewers. The first team included the authors and the second an external researcher with expertise in qualitative research. The objective of this assessment was to get familiar with the reports and evaluate their methodological rigor, as none of the studies had been excluded due to methodological quality^19^.

For each of the ten questions in the CASP, the following equivalents were determined: YES: 2, I DON'T KNOW: 1 and No: 0. The final score was the mean of the scores granted by each team of evaluators. Scores between 11 and 20 points were considered as lower risk of methodological bias, and scores inferior to 11 as higher risk of methodological bias.

Disagreements among the evaluators were solved through discussion or, when the research report did not comply with all evaluation criteria, access to the original text was sought, which happened in one case.

The average final score of the 14 studies was 14.5 or higher: one study reached 14,5^30^; four reached 15^27^ ^-^ ^29^ ^,^ ^34^; one 15.5^26^; another 16^33^; four reached 16.5^22^ ^-^ ^24^ ^,^ ^35^; two 18.5^25^ ^,^ ^31^ and, finally, one reached 19^32^. This demonstrates the rigor of the studies included in this meta-synthesis, guaranteeing the quality criteria required for systematic reviews^36^ and supporting the validity of the study results^19^.

To summarize the findings of this review, one of the foci suggested by Sandelowski and Barroso was adopted, which is the production of a qualitative meta-synthesis through constant comparative analysis^19^.

This process started with the reduction of findings from the primary sources through the manual extraction of 152 findings in an Excel matrix (2010), including the specific results the researchers presented as themes or metaphors and the participants' dialogues, in the original language and their translation to Spanish, with the respective page. This made it possible to reduce the mistaken extraction bias of the data from the primary sources^21^.

Based on reading and rereading, the findings were simplified manually, constituting 70 codes, grouped by similarities and differences. After the findings had been reduced through the coding process, they were regrouped into 64 subcategories, integrated in broader categories according to the relationship axis, until reaching the four final categories.

This coding and ranking process implied design, redesign and reformulation as the data were reviewed and their meaning emerged. The authors' joint analysis through meetings, discussion periods and particularly the secondary author's qualitative research expertise were key in this phase to be able to recognize, recontextualize, interpret the data and reach a consensus on the construction of the categories.

This facilitated the auditing of the analysis process^36^ and the reduction of bias in the recognition of the opposite results found^21^, through the inclusion of all evidence from primary sources and the exploration of the variations in the results.

To facilitate the understanding of the data and the understanding of the dynamics of the nurses' experience, eight visual models were formulated. Based on this process, the metaphor of the tightrope emerged as a useful resource to grant meaning to the data and favor the construction of the meta-theme (name proposed by McFarland and Wehbe)^37^_._

## Results

Among the 14 reports included, 13 are research articles^22^ ^-^ ^31^ ^,^ ^33^ ^-^ ^35^ and one a doctoral thesis^32^, published between 1993 and 2011. The studies were undertaken in nine countries: Great Britain, England, Ireland, Norway, Sweden, Canada, Colombia, Saudi Arabia and Australia. The designs employed included: exploratory (21.4%), descriptive-exploratory (28.6%), descriptive-interpretive (28.6%), phenomenological (7.1%), ethnographic (7.1%) and hermeneutic (7.1%).

Most studies were developed in hospital care scenarios (93%) and one in a home care context (7%). In total, 247 nurses participated in the 14 studies. Table 1 indicates the demographic characteristics of the nurses according to gender, age, country of origin and ethnic origin according to the reports. The item "not specified" indicates that this information was omitted in the primary study.

**Table 1:** Demographic characteristics of the nurses who participated in the primary studies according to gender, age, country of origin and ethnic origin. Bogotá, Colombia, 2014

Characteristic		Number	Percentage (%)
Gender			
	Women	102	41
	Men	32	13
	Not specified	113	46
Age			
	22-55	85	34
	Not specified	162	66
Country of origin			
	Australia	2	0.8
	Canada	9	3.6
	Ireland	1	0.4
	United Kingdom	1	0.4
	India	1	0.4
	Norway	16	6.5
	Sweden	52	21.1
	Colombia	20	8.1
	Not specified	145	58.70
Ethnic or cultural origin			
	White	40	16.2
	Black	3	1.2
	Latin	20	8.1
	Not specified	184	74.5

The nurses' characteristics according to education level, mean professional experience in years and cultural competency training are appointed in Table 2. Unfortunately, the latter two aspects were not determined precisely due to the lack of information in the primary studies (66% and 72% of the cases, respectively), representing a limitation for the synthesis.

**Table 2:** Characteristics of nurse participants in the primary studies according to education level, professional experience in years and cultural competency training. Bogotá, Colombia, 2014

Characteristic		Number	Percentage (%)
Education level			
	Auxiliary nurse	37	15
	Nursing professional	129	52
	Graduate	44	5
	Not specified	37	15
Mean professional experience (years)			
	<5	8	3
	6 to 10	40	16
	11 to 15	0	0
	16 to 20	23	9
	>21	12	5
	Not specified	164	66
Cultural competency training			
	Yes	70	28
	Not specified	177	72

**Figure 1:**
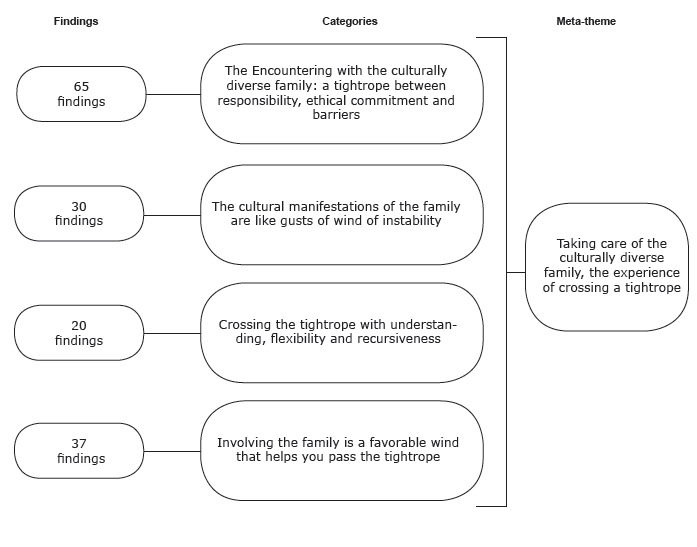
Categories and meta-theme resulting from the synthesis of the findings

Based on the interpretive integration of the findings from the 14 studies included, for categories emerged and one meta-theme, which represent the most significant result of this meta-synthesis process and facilitate the understanding of the nurses' experience in care delivery to culturally diverse families (Figure 1).

### Category 1: The Encountering with the culturally diverse family: a tightrope between responsibility, ethical commitment and barriers

The nurses' responsibility and ethical commitment in patient care make them take the first step in the encountering with the family. This meeting is developed amidst external and internal factors surrounding the care environment, called barriers, which makes the interaction developed during the encountering be neither static nor swinging and varying around the entire experience.

The external barriers are all difficulties for care delivery to the families caused by factors external to the nurses, primarily including communication difficulties: a) language differences^25^ ^-^ ^26^ ^,^ ^31^ b) confidentiality^24^ ^,^ ^29^ ^-^ ^30^ ^,^ ^33^ and c) cultural particularities in communication regarding gender distinctions or patriarchal attitudes in the families^27^, use of a child as translator^34^ or how to communicate with the health staff^29^.

Because of the presence of these barriers, the nurses perceive their responsibility to inform as demanding, as they acknowledge their duty to watch over the respect for the patients and families' right to be informed^27^, which gains complexity amidst the institutional policies about the use of family members as translators^23^.

Secondly, the institutional aspects that influence the delivery of excellent care to the family are considered part of the external barriers, determined by: a) lack of space in care areas to house all family members^28^ ^-^ ^30^ ^,^ ^32^ b) limitation of resources to offer food and accommodation to all family members^32^ and c) restricted time of the nurses to take care of the family^32^ ^-^ ^33^.

The presence of these barriers for the care experience has a negative impact, since the presence of the family is considered a problem or difficulty^22^ ^,^ ^29^ ^,^ ^32^, as it violates the institutional standards regarding visits and interferes in routine care procedures^25^ ^-^ ^26^ ^,^ ^29^ ^,^ ^32^, as one participant expressed: *The indigenous people used to arrive, as soon as they arrived they wanted us to attend to them in a consult and not only one but the entire family, if they are in ten they asked for ten consults, that way they were fine but if one was going to enter they all had to, so sometimes it was a problem for the consult, as they came from that far we could not have them wait that long* (E3P6, Nurse)^32^.

On the other hand, the presence of these barriers influences the emergence of internal barriers characteristic of the nurses, which appear when they experience a cultural shock in view of their own values and cultural beliefs, confronted with the values and cultural beliefs of the families^27^.

This confrontation raises obstacles for the advance towards a positive experience as the differences can cause conflicts. To give an example, some culturally diverse families' patriarchal opinion in conflict with being a woman in the nursing profession was reported as follows: *Her husband was the interpreter and before discharge he wanted a talk with the chief. I came, because I was the ward sister. He refused to talk to me, because I was a woman.* (Nurse)^26^.

The above leads to the negative perception of care for the families, manifested in: a) a negative image of the family, catalogued as an obstacle^25^ or a nuisance^32^ ^,^ ^35^ b) emotional reactions in the nurse, such as anguish^26^, stress^25^ ^-^ ^26^, tension^25^ ^-^ ^26^, uncertainty^26^, frustration^25^ and irritation^31^ and c) a distant and superficial relation with the family^29^.

### Category 2: The cultural manifestations of the family are like gusts of wind of instability

For the nurses, the family's cultural manifestations are different particular behaviors associated with culture, expressed in situations of disease, mourning, pain, acceptance or denial of a treatment. These manifestations are ranked as unexpected and different in view of their own culture as they can emerge at any time^28^ ^,^ ^30^ and take different forms: a) expressive or not expressive^26^ ^-^ ^30^ b) related to physical appearance and to the manifestation of customs^25^ ^,^ ^32^ c) related to the participation in care activities^27^ ^,^ ^33^ and d) associated with gender differences^27^ ^,^ ^32^, according to this participant: *The information is passed on by the husband, while in our culture it is different - when wives and mothers ask for information, they receive it. That can be a problem* (Nurse)^27^.

As a result of these cultural manifestations of the family, the balance the nurses maintain in encountering is threatened when no alternative answers are found. The background knowledge and experience are insufficient to approach the family, as reflected in this participant's expression: *As nurses we are engaged in, or we are used to helping grieving persons. But I didn't find this consolatory role in the unit with all those family members* (Nurse)^26^.

In addition, the nurses perceive that these manifestations affect the work environment and the other patients, when the alternative of cultural imposition emerges as the best answer. In that sense, one participant affirmed: *Family members stayed in the room after she died and talked very loudly. We had to shut the doors, in order to prevent the voices being heard in the corridor, because other patients were really scared* (Nurse)^26^.

### Category 3: Crossing the tightrope with understanding, flexibility and recursiveness

In response to the disequilibrium, the nurses start to consider additional tools that will help them gain skills to advance and overcome the instability that used to mark their meeting with the family: understanding, flexibility and recursiveness.

The understanding emerges when the nurses start to "put themselves in the families' place". Through understanding, they start to discover the family's characteristics and needs and to explain the manifestations that cause instability^22^ ^-^ ^24^ ^,^ ^26^ ^-^ ^27^.

On the other hand, flexibility is described as "having an open mind" to the family's needs. It implies being receptive without judging the family's attitudes, behaviors, which helps to understand the cultural differences^24^ ^,^ ^27^. To give an example, one participant affirmed: *You have to be flexible about the family coming to see the patient and not just stick to the visiting hours. Patients can feel very isolated if they can't talk to anybody, can't understand what's being done or what's being said around them* (Nurse)^24^.

Through these two tools, the nurses start to plan and implement alternative interventions, which are produced through recursiveness, considered as innovative forms of acting and solving the difficulties in dealing with the family.

To give an example, the nurses mobilize all resources within their reach to accommodate the families of indigenous patients^32^, create alternatives to preserve the beliefs, values, customs and popular care of the family or by participating in some of their family rites^25^ and facilitating a supportive climate^26^. On another occasion, they allowed the family members to take symbols and icons of cure^27^ or devised linguistic aids to facilitate the interaction with the family^26^.

One participant commented on an example of an alternative intervention based on recursiveness as follows: *Muslim women are usually covered up. When their shoulders are naked, we cover them by dressing them in a shirt back to front before the visitors arrive* (Nurse)^27^.

### Category 4: Involving the family is a favorable wind that helps you pass the tightrope

The tools of understanding, flexibility and recursiveness permitted reformulating the meaning of the experience positively. The nurses start to catalogue the help the family can offer them as "useful"^22^ ^,^ ^29^ ^,^ ^33^ ^-^ ^34^, through assistance^22^ ^,^ ^29^ ^,^ ^34^ in communication^22^ ^-^ ^24^ ^,^ ^31^, patient safety^22^ and support for the patient^29^ ^,^ ^31^, according to this participant: *I find the relatives very helpful. I am more appreciative of the help they give us in helping to wash the patient and also without forgetting with the interpreting. I don't know what we would do without them* (Staff nurse)^35^.

Feeling as if the family joined the purpose of taking care of the patient offers stability and triggers the construction and strengthening of a good relationship that offers benefits at three levels, for the patient, the nurse and the family: a) patient: benefits in terms of emotional and physical support^22^ ^,^ ^29^ ^,^ ^31^, comfort^24^, safety, trust^31^ ^,^ ^33^ and emotional, social and psychological wellbeing^25^ b) nurse: Overcome difficulties in communication^22^ ^-^ ^23^ ^,^ ^29^ ^,^ ^31^, enhancing their knowledge about the different cultures^22^ ^,^ ^24^ and strengthening the bond with the patient^22^ ^,^ ^28^ ^,^ ^31^ and c) family: Strengthening the trust in the nurse^26^ and delivering care or participating in decision making about the care of their loved one^25^ ^,^ ^29^ ^,^ ^33^.

### Meta-theme: Taking care of the culturally diverse family, the experience of crossing a tightrope

**Figure 2:**
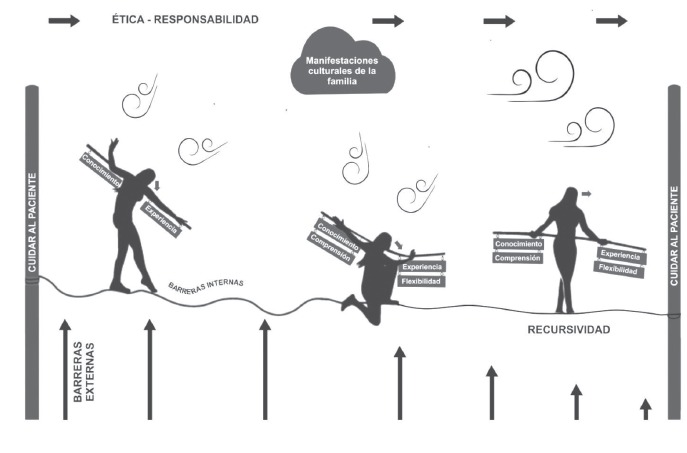
Nurses' experience taking care of culturally diverse families (TRAD: ethics-responsibility, cultural manifestations of the family, patient care, external barriers, knowledge, experience, internal barriers, experience, flexibility, recursiveness)

Engaging in the experience of crossing a tightrope and experiencing uncertainty due to the risk of falling in a marvelous and admirable, but also demanding and challenging act. That is the experience of the nurses in care delivery to culturally diverse families (Figure 2).

Like the tightrope, the encountering with the family is swinging and changing as the nurses advance and perceive their experience positive or negatively, depending on the interaction with the families and the different elements surrounding the care environment (Figure 2).

The nurses' first step in the encounter with the family is driven by the ethics and responsibility of patient care, motivated by the moral imperative of taking care as good as possible. These are the two hubs that sustain the encounter with the family.

The nurses start to feel like they are on a tightrope, because the encountering takes place amidst external and internal barriers, and they feel the family's cultural manifestations as gusts of winds. Their perspective focuses downwards, letting themselves be affected by the barriers and difficulties (Figure 2), which makes them feel unstable and causes a negative perception of the experience.

Consequently, care for the family is perceived as an obligation or a requirement that is difficult to respond to. The tools they have counted on and which they take along their balance beam: knowledge and background experience (Figure 2) are insufficient to approach the culturally diverse family, making them respond with cultural imposition.

Nevertheless, amidst the instability, the nurses take a break (Figure 2) and start to consider additional tools to approach the family: understanding and flexibility, which are added to their balance beam and offer them stability (Figure 2).

Thus, they start to advance more safely through the responses based on recursiveness, their perspective turns forwards and their perception of height decreases progressively (Figure 2), which allows them to perceive those elements that used to be against them as now in favor.

Finally, the nurses start to value the benefits of taking care of and with the family, strengthening their care relationship, which favors the advance towards a positive perception of the experience.

## Discussion

This study was aimed at interpreting and summarizing the findings of qualitative studies to understand the experiences of nurses in care for culturally diverse families. The interpretive effort in the meta-synthesis process offered new meanings for the findings reported in the primary studies, transcending the explanation of the dynamics and meanings of the nurses' experience.

The visual representation of the synthesis as a tightrope (Figure 2) turned into a powerful rhetorical resource to interpret the experience not only more understandably for the readers, but also to sustain the validity of the results, as this type of resource in meta-syntheses reveals the relations discerned and comparisons the authors made^19^.

On the one hand, the findings of this meta-synthesis suggest that the encounter with the culturally diverse family is relational, dynamic and swinging, because it permits intercultural interaction with the family^2^ and constitutes the central platform where meanings are constructed^38^.

What is particular about the encountering is the characteristic of being mediated by patient care, in which the approach of the family takes place amidst the view of the family as the patient's context, despite the belief that the approach of the family as the care unit has always been a focus of interest for nursing^17^.

This evidences the prevalence of the rupture between "what should be" and the "actual practice" of family nursing^39^, amidst systems and organizations that make it difficult to include the families in care, all the more in the hospital contexts that are predominant in most of the studies included in this meta-synthesis.

The nurses' efforts to approach the families, motivated by the ethical responsibility of care, is restricted not only by the presence of external barriers related to the communication difficulties, the lack of time for care, the lack of space for the visits and resources for the families, but also by the presence of internal barriers related to the impact of cultural differences.

It is not surprising that this meta-synthesis reaffirms the nurses' difficulty to respond to the family requirements when there are cultural differences that make this task more complex. The novelty lies in the effect of these factors causing permanent tension, generating a negative perception of the experience, manifested in the appearance of feelings, challenging emotions and conflicts and resulting in a distant and superficial relation with the family.

On the other hand, the findings in this meta-synthesis demonstrate the appearance of some elements of cultural competence in response to the instability in care for culturally diverse families. When the nurses start to attribute meaning to the differences by acknowledging the family's and their own needs through understanding and flexibility, they find alternative responses within the possibilities and resources they have access to.

Thus, understanding and flexibility in the light of Campinha-Bacote's Theory of Cultural Competence^2^ reflect the cultural awareness, while the other elements: desire, knowledge and cultural skills^2^ were manifested incipiently in the experience through the alternatives that were produced amidst the recursiveness.

Unfortunately, the omission of information in the participants' reported characteristics in the research reports, in this case concerning the nurses' cultural competency background, was a limitation that hampered the depth of this analysis. It would have been preferable to determine whether these nurses possessed cultural competency knowledge that allowed them to produce novel alternative responses or whether spontaneous solutions emerged amidst the instability.

Despite this limitation, this synthesis managed to demonstrate that these elements, associated with cultural competency, contribute to the positive reformulation of the meaning of the experience. Although the tension prevails, thanks to these elements, the perception of the experience gains a new understanding that permits valuing the benefits of engaging the family in care.

Based on this evidence, it is concluded that the meaning of the nurses' experience in care delivery to culturally diverse families is dynamic and can move from a positive to a negative perception or vice-versa, according to the interaction with the family, the factors in the care context and the tools of cultural competency.

### Implications for nursing research and practice

This meta-synthesis was proposed to have an international range, including a culturally diverse sample and different care contexts. Nevertheless, the inclusion of a single study undertaken in Latin America^32^ evidences the lack of empirical literature on this theme in our background.

In addition, the prevalence of hospital-based scenarios over communities shows the need to further investigate this study phenomenon in other nursing care contexts with multicultural populations.

On the other hand, it is fundamental to broaden the discussion by focusing future research efforts on the study of cultural competency and exploration from the perspective of the families' experience, which would permit furthering the understanding of the potential intervention strategies directed at culturally diverse families, from the viewpoint of the care providers as well as the objects of their care.

The limitations in the reports of the studies included in this review demonstrate that improving the quality of the research reports is fundamental, with greater emphasis on the participants' reports and on the design, which continues being a weak point in qualitative reports.

In practice, these study results provide a baseline for health organizations to focus most of their intervention efforts on the external barriers, implementing mechanisms like: a) the establishment of appropriate and competent linguistic services; b) the destination of more resources and the creation of spaces to accommodate the family members and c) the evaluation of the time distribution for the performance of professional activities.

The nursing professionals, in turn, need to focus on the internal barriers: a) heeding the recognition, understanding and respect for the cultural particularities that characterize the people they take care of and b) favoring intercultural education spaces.

Finally, this study reaffirms the need to train nursing professionals with cultural competences at the different formal education levels (basic and specialized) and to adapt curricula, with a view to favoring spaces that stimulate knowledge and skills in care delivery to culturally diverse populations and to gaining positive care experiences.

## Conclusion

The summary of the findings of 14 qualitative studies permitted understanding the nurses' experience in care delivery to culturally diverse families as a gratifying and admirable, but also demanding and challenging experience, in view of the complex approach of the family unit, the encounter with cultural differences and the ethical responsibility of care that gains complexity when it enters the family sphere.

This new understanding from the professionals' perspective revealed the permanent tension the nurses experience in care to families from different cultures and its consequences for the perceived meaning of the experience and for the care relationship.

The evidence of this review opens new horizons in knowledge on family and cross-cultural nursing, offering a more reliable base for decision making in the approach of culturally diverse families and revalidating the need for nurses who are prepared to respond to the demands of a multicultural world, not only due to the impact of providing culturally adapted care, but also because of the satisfaction and positive perception in the care experience.
